# Balancing AI and human insights in scientific discovery: Challenges and guidelines

**DOI:** 10.1016/j.xinn.2025.101144

**Published:** 2025-09-30

**Authors:** Ricardo Vinuesa, Pilar Manchón, Sergio Hoyas, Javier García-Martínez

**Affiliations:** 1 Department of Aerospace Engineering, University of Michigan, Ann Arbor, MI 48109, USA; 2 FLOW, Engineering Mechanics, KTH Royal Institute of Technology, Stockholm 100 44, Sweden; 3 Google Research, 1600 Amphitheatre Parkway, Mountain View, CA 94043, USA; 4 Instituto Universitario de Matemática Pura y Aplicada, Universitat Politècnica de València, València 46022, Spain; 5 Molecular Nanotechnology Lab, Department of Inorganic Chemistry, University of Alicante, Alicante 03080, Spain

## Main text

Recent advances in large language models (LLMs) have enabled machines to integrate web search, code execution, data analysis, decision-making, and even laboratory experimentation, as done in chemical discovery using the “co-scientist.”^1^ This artificial-intelligence (AI)-driven platform represents a pivotal moment in the evolution of systems. By using LLMs such as GPT-4 and Claude, co-scientists can autonomously design, plan, and execute complex chemical experiments based on simple natural-language prompts. Their capability lies in the ability to interpret plain-language requests, perform extensive data searches, synthesize information, and autonomously operate laboratory equipment through robotic application programming interfaces (APIs).

These capabilities can offer significant benefits for scientific writing and research proposal generation: for instance, they can assist researchers in articulating ideas more effectively, reducing language barriers, and streamlining administrative aspects of proposal preparation. In this context, the integration of AI into scientific proposal writing has accelerated in recent months. In particular, Google’s recent launch of the AI co-scientist, a virtual collaborator to assist scientists based on Gemini 2.0, has the potential to revolutionize the generation of novel hypotheses and research plans.^2^ Early collaborations with institutions such as Stanford University and Imperial College London have demonstrated its ability to independently hypothesize novel gene transfer mechanisms and suggest potential treatments for diseases such as liver fibrosis. Similarly, platforms such as Future House (https://www.futurehouse.org/) are emerging to further automate the scientific discovery processes, indicating a broader trend toward AI-driven research methodologies.

In fact, the adoption of LLMs in research is accelerating rapidly. A recent *Nature* survey reported that 81% of researchers have used AI tools like ChatGPT in their work.^3^ This trend highlights the urgent need to critically assess how these tools influence scientific workflows. This assessment is not occurring at the rate of change being produced.

The integration of AI into all stages of discovery, from hypothesis generation to interpretation of results, also raises significant risks and ethical concerns, including funding allocation. For example, the efficiency of AI-driven research may deprioritize projects that rely on intuition, reflection, and longer timelines, potentially stifling novelty and ambition.

AI use also opens avenues for misconduct, such as data fabrication or biased designs, without proper oversight. Moreover, it can manipulate public opinion and scientific narratives. A historical parallel is how the sugar industry shifted blame for health issues to dietary fat through manipulated research and lobbying, with long-term consequences in the United States. With LLMs able to generate persuasive content at scale, similar tactics could now be deployed more efficiently. In addition, control of advanced tools by a few companies or countries could monopolize discovery, using it as a means of dominance.

Another important point is lateral thinking. LLM-generated ideas show some creativity, complementing human contributions. Yet, the proportion of breakthrough discoveries has declined: a 2023 study analyzing 45 million papers and 3.9 million patents found a marked drop in the “disruption index” across disciplines since the 1940s.^4^ This is partly due to incentives favoring incremental projects. If LLMs are widely adopted for proposal writing, they may reinforce this trend by prioritizing ideas aligned with existing literature, limiting cross-disciplinary innovation. Trained on past research, they reflect historical biases and conventional paradigms, restricting radically new concepts. They are designed to fill gaps between known facts, not to generate truly novel insights.

To draw an analogy: imagine the time just after Newton had developed calculus and mechanics. AI could refine and scale this framework but would lack the insight of an Einstein or a Planck, capable of revolutionizing physics through relativity and quantum theory. AI might push classical mechanics to its limit, yet it would never confront questions such as whether Schrödinger’s cat is alive or dead.

An even more concerning prospect is the potential delegation of both proposal writing and evaluation to LLMs, with human scientists relegated to executing research projects shaped by AI. This scenario is not hypothetical: a survey^5^ showed that 19% of researchers already use LLMs in the peer-review process, and 7%–17% of reviews in recent AI conferences were significantly modified using these tools. This would create a self-reinforcing feedback loop where AI-generated proposals are assessed by AI-driven review systems, further entrenching incremental research trends. Without deliberate intervention, this scenario could sideline human creativity and intuition in scientific idea generation, reducing the likelihood of transformative scientific breakthroughs.

**Figure 1:**
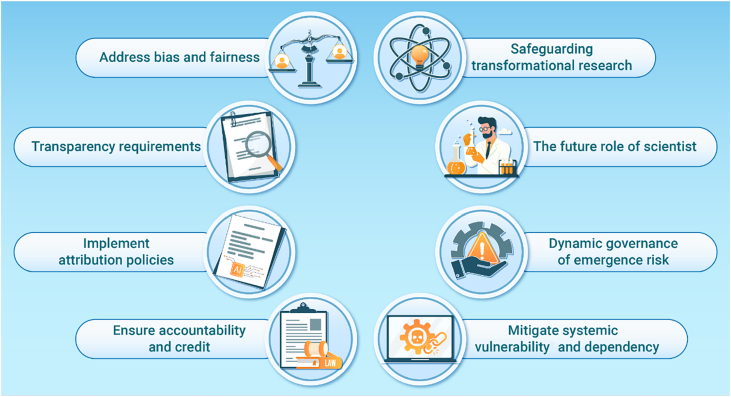
Visual summary of guidelines for the ethical use of AI in the design of scientific proposals

Given these potential consequences, it is essential to carefully consider the ethical implications of integrating LLMs into the research proposal process. Ensuring that AI remains a tool for augmentation rather than automation requires proactive measures to address biases, promote transparency, and maintain human oversight. The following ethical guidelines (see Figure 1) outline strategies to mitigate risks while preserving the integrity and transformative potential of scientific research.

(1) Address bias and fairness: bias and fairness must be systematically addressed to avoid reinforcing inequities in research. Regular audits of outputs should be conducted to detect and mitigate bias. Interdisciplinary teams can provide broader perspectives, while external review mechanisms are needed to capture biases not apparent to domain experts. Incorporating diverse viewpoints and rigorous oversight is essential for equitable AI-assisted inquiry.
(2) Transparency requirements: transparency is essential for maintaining trust. Researchers should carefully document input parameters, training data, and decision pathways used by LLMs. Open-access repositories must allow tracing of datasets and algorithms that shape AI hypotheses. Model-explainability techniques are required so that discoveries can be understood, interrogated, and reproduced by human experts. Without transparency, researchers risk adopting ideas without knowing their origins or limitations.
(3) Implement attribution policies: attribution policies must be clearly defined to ensure acknowledgment of source materials and address biases embedded in training data. The evolving nature of authorship should be recognized if AI functions more as a collaborator than a tool. A new framework for intellectual property is necessary to distinguish human-driven from AI-generated innovations. Without clear guidelines, questions of ownership and contribution could undermine confidence in AI-based research.
(4) Ensure accountability and credit: accountability remains a cornerstone of scientific integrity. AI-generated content must undergo rigorous validation, including mandatory cross-checking of references and data. Human scientists must play an active role in validating and interpreting outputs before publication. Professional and legal responsibility should also be clarified to prevent the unchecked spread of erroneous or unethical research. AI should support human work, not become an unregulated driver of discourse.
(5) Safeguarding transformational research: the risk of LLMs reinforcing incremental projects at the expense of breakthroughs must be addressed. Dedicated grant programs should support high-risk, interdisciplinary work that may not align with conventional trends. Human-centric review processes must remain in place to ensure that unconventional ideas are considered. Training programs should prepare scientists to evaluate AI proposals critically and counteract biases favoring incremental research. Such measures are essential to foster truly innovative discoveries.
(6) The future role of scientists: as AI becomes embedded in research, scientists must cultivate intuition, ethical judgment, and long-term vision. Education should emphasize AI literacy, critical evaluation, and ethics. Research cultures must balance efficiency with curiosity, ensuring human insight guides discovery. AI should not replace expertise but enhance it, allowing focus on higher-order questions and creative problem-solving
(7) Dynamic governance for emergent risks: because LLMs evolve rapidly, static regulation is insufficient. Governance must be dynamic, able to monitor and respond to emerging risks. Adaptive oversight, regular assessments, and regulatory sandboxes with academia, industry, and policy-makers are needed. Real-time auditing and scenario simulations can capture unexpected outcomes and guide timely ethical and operational adjustments.
(8) Mitigate systemic vulnerability and dependency: widespread integration of LLMs into research workflows may create systemic dependencies on proprietary models and centralized infrastructures. Such concentration of power introduces fragility into the global research enterprise. Open-source alternatives and institutional redundancy plans should be supported. Efforts must also prevent vendor lock-in and promote diversity in infrastructure, data, and training sources. Building resilient, interoperable, and ethically aligned ecosystems is essential to preserving autonomy and scientific pluralism.

These guidelines should build on existing open-science initiatives such as the FAIR principles, reproducibility standards and open data policies, ensuring interoperability and transparency. By adhering to these guidelines, the scientific community can harness LLM’s potential to generate research proposals while maintaining research integrity and ethical standards. However, proactive measures must be taken to counteract the risks of increased reliance on AI-generated research, ensuring that human creativity and intuition remain at the heart of scientific progress. Without such safeguards, there is a real danger that the increasing use of AI in research will contribute to a decline in transformational breakthroughs, ultimately limiting scientific advancement.

## Declaration of interests

The authors declare no competing interests.
